# Distinct complication profiles: a comparative study of Ethiopian and non-Ethiopian adults with type 1 diabetes

**DOI:** 10.3389/fendo.2025.1664230

**Published:** 2025-10-17

**Authors:** Alena Kirzhner, Amir Bashkin, Hefziba Green, Haitham Abu Khadija, Shay Teitlboim, Meital Zikry Deitch, Mohammad Alnees, Merav Greenstein, Tal Schiller

**Affiliations:** ^1^ Department of Internal Medicine A, Kaplan Medical Center and Faculty of Medicine, Hebrew University of Jerusalem, Rehovot, Israel; ^2^ Department of Diabetes, Endocrinology and Metabolism, Galilee Medical Center and Azrieli Faculty of Medicine, Bar-Ilan University of Safed, Nahariyya, Israel; ^3^ Department of Cardiology, Kaplan Medical Center and Faculty of Medicine, Hebrew University of Jerusalem, Rehovot, Israel; ^4^ Pharmacy Services, Kaplan Medical Center Faculty of Medicine, Hebrew University of Jerusalem, Rehovot, Israel; ^5^ Intensive care unit, Kaplan Medical Center, and Faculty of Medicine, Hebrew University of Jerusalem, Rehovot, Israel; ^6^ Harvard Medical School, Postgraduate Medical Education, Global Clinical Scholar Research Training Program, Boston, MA, United States; ^7^ Merav Statistical Consulting, Kaplan Medical Center, Rehovot, Israel; ^8^ Institute of Endocrinology, Diabetes and Metabolic Disease, Wolfson Medical Center, Holon, Israel; ^9^ Affiliated to the Gray Faculty of Medical and Health Sciences, Tel Aviv University, Tel Aviv, Israel

**Keywords:** type 1 diabetes (T1D), microvascular complications of diabetes, macrovascular complications of diabetes, Ethiopian ethnicity, diabetes mellitus

## Abstract

**Aims:**

Ethiopian ethnicity is linked to a higher risk of diabetes, yet data on disease characteristics and complications in Ethiopians with type 1 diabetes (T1D) are limited. This study aimed to assess clinical features and complication rates in Ethiopian versus non-Ethiopian T1D patients.

**Methods:**

This population-based retrospective cohort study included all patients insured in Clalit Health Services (CHS) who were considered to have T1D according to study criteria between January 1, 2000, and December 31, 2022. Patients were followed until December 31, 2023, for the development of composites of microvascular and macrovascular complications.

**Results:**

Among 12,759 T1D patients, 672 (5.3%) were Ethiopian, and 4,375 (34%) were diagnosed before age 18. The mean age was 30.4 years, 54% were male, and the mean BMI was 25.4 kg/m². Average follow-up was 10.9 years. In multivariable Cox regression models, Ethiopian ethnicity was an independent risk factor for microvascular complications (hazard ratio [HR] 1.325; 95% CI 1.124–1.563; p = 0.001) but was associated with a lower risk of macrovascular complications (HR 0.606; 95% CI 0.425–0.863; p < 0.001).

**Conclusions:**

The striking differences in diabetes-associated complications underscore the need for ethnic-specific and population-specific follow-up, therapeutic, and preventive approaches for T1D patients.

## Highlights

• What is already known about this subject?
Significant gaps exist in understanding disease characteristics and complication development among ethnic- and population-specific patients with type 1 diabetes (T1D).
• What is the key question?
What is the clinical phenotype and rate of microvascular and macrovascular complications in patients with T1D of Ethiopian versus non-Ethiopian ethnicity?
• What are the new findings?
Ethiopian ethnicity emerged as a significant risk factor for the development of microvascular complications, while being associated with a lower incidence of macrovascular complications.
• How might this impact clinical practice in the foreseeable future?
Highlighting the need for ethnic- and population-specific therapeutic and management approaches for T1D.

## Introduction

Type 1 diabetes (T1D) is an autoimmune disease with a rising incidence and prevalence, increasing at a rate of approximately 2–3% per year (1). Poor glycemic control is strongly associated with microvascular and macrovascular complications; however, it does not fully account for the variability in disease severity and complication rates among individuals.

Additional factors have been linked to the development of complications, including diabetes duration, dyslipidemia, hypertension, family history, and ethnicity (2). Adding to the complexity of T1D progression, recent research has introduced the concept of endotypes, which categorize the disease into subgroups based on factors such as age, HLA genotyping, and the first autoantibody detected (3, 4). However, most existing data are derived from Caucasian populations, with limited research on ethnic minorities.

Ethnicity has been suggested as an important factor in complication development and progression, yet data remain scarce. Several studies, to date, suggest that the rate of microvascular and macrovascular complications differs between different ethnicities (5, 6).

The Ethiopian population in Israel is comprised of immigrants and descendants of Ethiopian Jews who immigrated to Israel in the 1980s and 1990s. Since then, immigration has continued on a smaller scale. As of the end of 2023, according to the Israeli Central Bureau of Statistics, people of Ethiopian descent comprised less than 2% of the entire Israeli population (171,000 Ethiopians and approximately 10 million of the general population) (7).

The prevalence of diabetes among Ethiopian immigrants to Israel has changed drastically from 0.4% in the 1980s to approximately 17%, compared with 8.4% of the general Israeli population (8, 9). Suggested explanations included different lifestyles, nutrition, and lower socioeconomic status.

Focusing on T1D, Zung et al. found a high incidence of T1D due to ongoing exposure of genetically predisposed immigrants from Ethiopia to diabetogenic environmental factors, eventually leading to a high incidence of overt diabetes in this ethnic group (10). To date, we have been unable to find specific data on T1D phenotype and complications among the Ethiopian population in Israel.

The vast immigration from Ethiopia to Israel in recent decades has created unmet gaps in our understanding of clinical phenotype, complications development, and disease progression in this population; thus, understanding the natural history is critical. This study aimed to characterize the clinical phenotype and rate of microvascular and macrovascular complications in Israeli patients with T1D of Ethiopian versus non-Ethiopian ethnicity.

## Materials and methods

This population-based retrospective cohort study is based on data from insured members of Clalit Health Services (CHS), Israel’s largest integrated healthcare service system, covering half of the population. CHS has a comprehensive healthcare data warehouse, integrating demographic information, hospital and community medical records, laboratory and imaging information, pharmaceutical records, and Ministry of the Interior Statistics (11). CHS membership includes a relatively large proportion of minorities (27% vs. 21%) compared to the general population (12). The study and data usage were approved by Clalit’s Institutional Review Board and the local Institutional Review Board.

**Figure 1:**
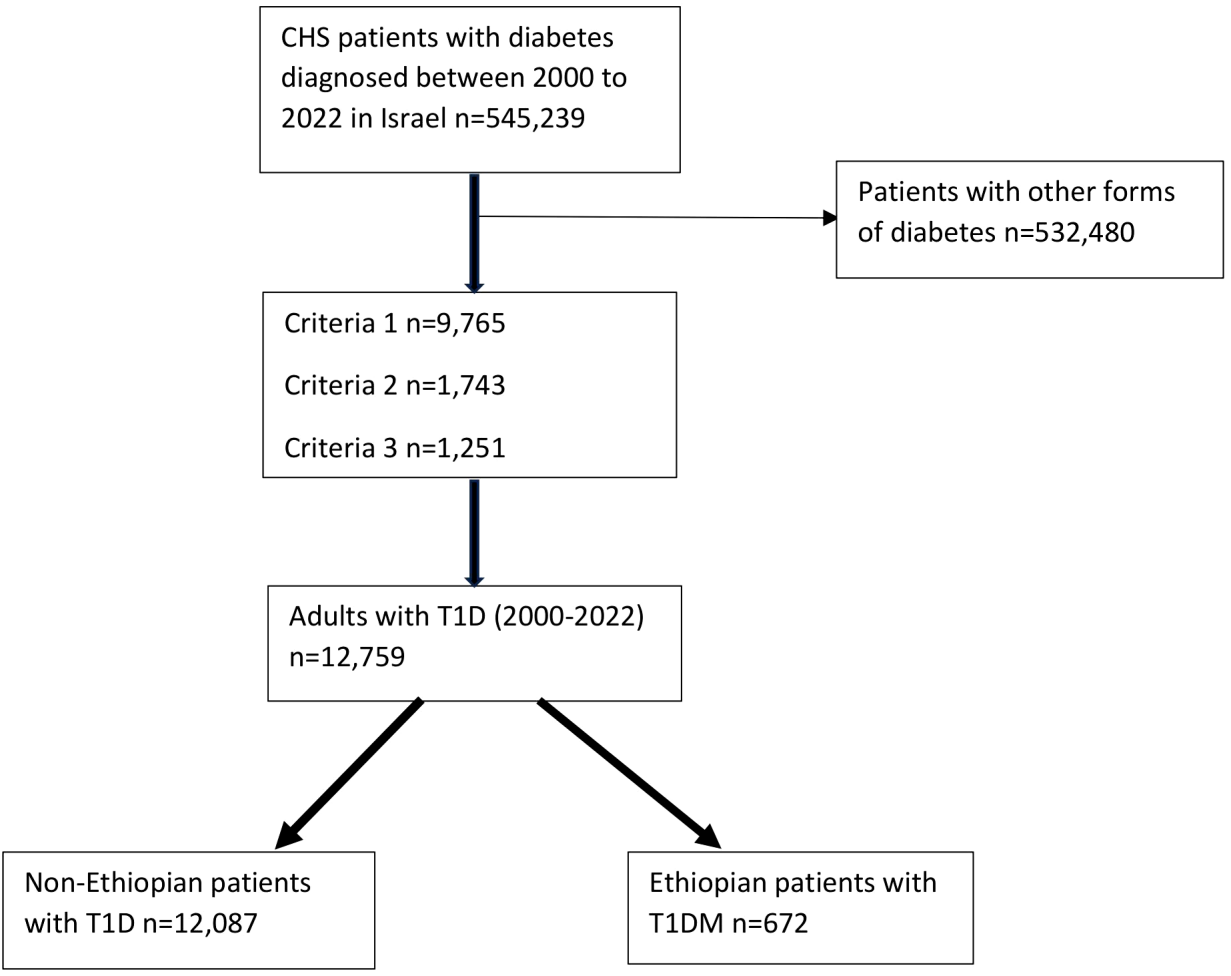
A flow chart showing the recruitment of the subjects. CHS: Clalit Health Services; T1D: Type 1 diabetes. Criteria 1: Patients at any age with positive autoantibodies, including anti-GAD and/or anti-islet cell antibodies or a decreased C-peptide with a blood level of below 0.2 nmol/L or equivalent results six months before until one year after a diabetes diagnosis; Criteria 2: Patients diagnosed with diabetes at the age of < 18 years and more than two purchases of any fast-acting insulin (Actrapid, or Apidra (Glulisine), or Humalog (Lispro), or Novorapid (Aspart)) six months before until one year after a diabetes diagnosis; Criteria 3: Patients diagnosed with diabetes between 18-40 years and more than three purchases of fast-acting insulin (as above), six months before until one year after a diabetes diagnosis.

The index date for each patient was defined as the first diagnosis of T1D. All patients who met the study criteria for T1D between January 1, 2000, and December 31, 2022, were included in the analysis if they had reached the age of 18 years or older by December 31, 2022 (**Figure 1**).

The follow-up period for diabetes complications spanned from the time of T1D diagnosis (index date) to the date of any diabetes complications, death, end of CHS membership, or December 31, 2023, whichever occurred first.

Since a diagnosis of T1D is not specifically recorded, a set of criteria was used for T1D diagnosis.

Diabetes was diagnosed using ICD-10 codes E10 and E11 (**Supplementary Table 1**). Additionally, any one of the following criteria was mandatory for a patient to be considered as having T1D:

1. Patients at any age with positive autoantibodies, including anti-GAD and/or anti-islet cell antibodies or a decreased C-peptide with a blood level of below 0.2 nmol/L or equivalent results six months before until one year after a diabetes diagnosis, OR
2. Patients diagnosed with diabetes at the age of < 18 years and more than two purchases of any fast-acting insulin (Actrapid, or Apidra (Glulisine), or Humalog (Lispro), or Novorapid (Aspart)) six months before until one year after a diabetes diagnosis, OR
3. Patients diagnosed with diabetes between 18-40 years and more than three purchases of fast-acting insulin (as above), six months before until one year after a diabetes diagnosis.

Insulin autoantibodies (IAA) were not included in the analysis as we could not verify the timeline of insulin exposure to IAA measurement. Zinc-8 transporter antibodies were not considered as they were not available during the study period.

The following exclusions were used (according to ICD-10 codes): 1. A diagnosis of gestational diabetes; 2. Patients with neonatal diabetes mellitus; 3. Patients with diabetes due to an underlying condition (post-pancreatectomy diabetes mellitus, pancreatitis, postprocedural diabetes mellitus, and other diseases of the pancreas).

Ethnicity was categorized as Ethiopian versus non-Ethiopian. People were considered of Ethiopian ethnicity if the recorded country of birth was Ethiopia for either them or one of their parents. The non-Ethiopian group included all other ethnic origins (mainly Caucasian Israeli population and mixed minorities).

After establishing the study cohort, the following diabetic complications were extracted from the electronic medical records (EMR), according to ICD-10 codes, and two composites for microvascular and macrovascular complications were assessed:

A microvascular composite endpoint included retinopathy, nephropathy, and neuropathy and a macrovascular composite endpoint included ischemic heart disease (IHD), myocardial infarction (MI), chronic heart failure (CHF), peripheral vascular disease (PVD), carotid artery disease, cerebrovascular accident (CVA), and transient ischemic attack (TIA). Only events occurring after the diagnosis of T1D were included in the composite outcomes to avoid misclassification of prevalent disease as incident.

Additional diagnoses were recorded if they ever appeared in the EMR, including alcohol consumption, hypertension, hyperlipidemia, obesity, and metabolic-associated liver disease according to ICD-10 codes.

Socioeconomic variables measured at the index date included age, sex, immigrant status, and socioeconomic status (SES): low, medium, high. SES can be determined at the clinic level per the designation of each member’s primary care clinic based on census designations from the Israeli Central Bureau of Statistics.

Weight, height, and body mass index (BMI) were determined based on the closest documented value from the index date to five years after diagnosis. Blood pressure (BP) measurements were determined based on the closest available measurement to the index date, spanning from a year before to a year after the index date.

Smoking status was categorized as current or former smoker if any smoking history was recorded and never smoking if there was no record of any smoking history.

Laboratory data, including glycated hemoglobin (HbA1c), lipid profile, and creatinine, were recorded as the maximal value closest to the index date, from a year before to a year after. Similarly, the urine microalbumin to creatinine ratio was recorded as the maximal value closest to the index date, from a year before to two years after.

To maximize statistical power and external validity, we opted against matching or propensity score methods, which would have reduced our sample size and potentially limited generalizability to the broader T1D population. Instead, we employed multivariable Cox regression models adjusting for key confounders identified through clinical expertise (age at diagnosis, sex, baseline HbA1c, BMI, and Ethiopian ethnicity). While this approach may be subject to residual confounding, it preserves the heterogeneity of our cohort and allows for direct estimation of covariate effects.

### Statistical analysis

Categorical and nominal variables were reported as frequencies and percentages. Continuous variables were assessed for normality using the Shapiro-Wilk test. Comparisons between continuous variables were performed using the independent samples t-test for normally distributed data and the Mann-Whitney U test for non-normally distributed data. Associations between categorical and nominal variables were analyzed using Pearson’s chi-square (χ²) test or Fisher’s exact test, as appropriate.

Survival analyses were conducted using Cox proportional hazards regression models to assess the association between predictor variables and the time to development of complications. Two separate models were constructed: one for microvascular complications and one for macrovascular complications. In each analysis, individuals with a documented diagnosis of the respective complication type at baseline were excluded to focus on incident events. Each participant contributed only once to each model. Models were adjusted for age, sex, ethnicity, BMI, and HbA1c. Hazard ratios (HRs) presented in the results represent adjusted estimates from the multivariable models unless otherwise specified.

To address potential age confounding in the macrovascular models, we re−estimated Cox proportional hazards using attained age as the time scale, implementing left truncation at age at cohort entry and using attained age at event/censoring as survival time. The primary attained−age model adjusted for sex, BMI, and HbA1c; a sensitivity model additionally adjusted for smoking status, obesity, and SES.

To further disentangle ethnicity effects from baseline risk differences, we re−fit the macrovascular Cox models using attained age as the time scale within strata of age at cohort entry (18–39, 40–59, ≥60 years; the <18 group was underpowered and is not interpreted) and within strata of a baseline cardiovascular risk profile defined as the count of classical risk factors (hypertension, hyperlipidemia, obesity: 0, 1, ≥2). Within each stratum, we estimated a primary model adjusting for sex, BMI, and HbA1c, and an extended model additionally adjusting for smoking and SES; risk−factor indicators that were constant by design in a stratum were excluded by the software. We also conducted a restriction analysis for age at entry ≥40 years.

A backward elimination approach was employed to systematically select significant variables, based on likelihood ratio chi-square tests. Model performance and multicollinearity were evaluated using variance inflation factors (VIF).

For handling missing data, we employed a complete-case analysis approach in all multivariable models. Variables with moderate levels of missingness were retained in the analyses, while variables with substantial missingness were excluded from the models. The resulting analytic sample sizes and number of events are reported for each model specification.

Statistical significance was determined at a two-tailed P-value of less than 0.05. All statistical analyses were executed using IBM SPSS Statistics for Windows, version 28.0.

## Results

### Study cohort characteristics

The cohort included 12,759 patients with T1D, of whom 672 (5.3%) were of Ethiopian ethnicity. 4,375 (34%) T1D patients were diagnosed before the age of 18 years, while 3,780 were diagnosed after the age of 40 years. The mean follow-up time was 10.9 ± 6.1 years. The mean age at diagnosis for the entire cohort was 30.4 ± 18.9 years, and 54% were male. The mean BMI was 25.4 ± 5.5 kg/m^2^, and the mean systolic BP and diastolic BP were 118.4 ± 15.5 mmHg and 72.2 ± 10.3 mmHg, respectively. Patient characteristics are depicted in **Table 1**.

**Table 1:** Characteristics of the study cohort: overall and stratified by non-Ethiopian and Ethiopian ethnicity.

Characteristics*	Total (n=12,759)	Non-Ethiopian (n=12,087)	Ethiopian (n=672)	P value**
Age at diagnosis T1D, years	30.4 ± 18.9	30.5 ± 19.0	29.0 ± 17.3	0.083^a^
Males	6835 (54)	6437 (53)	398 (59)	**0.003** ^b^
BMI, kg/m^2^	25.4 ± 5.5	25.5 ± 5.5	23.3 ± 4.1	**<0.001** ^a^
Missing	2,709 (21)	2,534 (21)	175 (26)	
Systolic BP, mmHg	118.4 ± 15.5	118.4 ± 15.5	116.9 ± 15.2	0.084^a^
Missing	5,031 (39)	4,802 (40)	229 (34)	
Diastolic BP, mmHg	72.2 ± 10.3	72.2 ± 10.4	72.1 ± 9.7	0.591^a^
Socioeconomic status:				**<0.001** ^b^
Low	6509 (51)	5993 (50)	516 (77)	
Medium	1947 (15)	1928 (16)	19 (3)	
High	3228 (25)	3114 (26)	114 (17)	
Missing	1075 (8)	1052 (9)	23 (3)	
Smoking status				**0.005** ^b^
Never	7544 (59)	7129 (69)	415 (73)	
Current	1995 (16)	1887 (18)	108 (19)	
Former	1402 (11)	1354 (13)	48 (8)	
Missing	1,818 (14)	1,717 (14)	101 (15)	
Alcohol use	218 (2)	183 (2)	35 (5)	**<0.001 ^b^**
Chronic diseases before, at, or after the index date				
Obesity	4565 (36)	4439 (37)	126 (19)	**<0.001 ^b^**
Hypertension	3363 (26)	3221 (27)	142 (21)	**0.002 ^b^**
Hyperlipidemia	2223 (17)	2147 (18)	76 (11)	**<0.001 ^b^**
Metabolically associated liver disease	923 (7)	879 (7)	44 (7)	**0.480 ^b^**
Laboratory results at the index date				
HbA1c, %	9.8 ± 2.9	9.8 ± 2.8	10.4 ± 3.0	**<0.001 ^a^**
Missing	1,761 (14)	1,687 (14)	74 (11)	
Total Cholesterol, mg/dL	196.2 ± 53.7	196.5 ± 53.9	191.8 ± 49.1	**0.025 ^a^**
Missing	1,218 (10)	1,159 (10)	59 (9)	
LDL-Cholesterol, mg/dl	115.6 ± 38.9	115.7 ± 39.0	113.5 ± 37.7	0.078 ^a^
Missing	2,948 (23)	2,808 (23)	140 (21)	
Triglycerides, mg/dL	194.4 ± 232.4	195.1 ± 235.0	181.9 ± 179.7	0.165 ^a^
Missing	1,297 (10)	1,233 (10)	64 (10)	
Creatinine, mg/dL	0.8 ± 0.5	0.8 ± 0.5	0.7 ± 0.2	**<0.001 ^a^**
Missing	1,110 (9)	1,059 (9)	51 (8)	
eGFR, ml/min/1.73m^2^	115.1 ± 19.2	114.6 ± 19.3	123.1 ± 15.5	**<0.001 ^a^**
Missing	5,898 (46)	5,633 (47)	265 (39)	
Albumin/creatinine ratio, mg/g	39.1 ± 152.4	38.9 ± 152.1	42.7 ± 157.6	0.443 ^a^
Missing	6,947 (54)	6,580 (54)	367 (55)	

T1D, Type 1 diabetes; BMI, Body mass index; BP, blood pressure; HbA1c, Hemoglobin A1c; eGFR, Estimated glomerular filtration rate; LDL, Low-density lipoprotein; SD, Standard deviation.

*Data presents mean ± SD or n (%). **Patients non-Ethiopian vs Ethiopian; P-value is significant at <0.05.

a Two-sample t test.

b Chi-Square Test (χ²). Bold means the value has a statistical significance.

When categorized according to ethnicities, patients of Ethiopian ethnicity were more likely to be male, of lower SES, and leaner. They were less likely to receive a diagnosis of hypertension or hyperlipidemia. Additionally, Ethiopian participants had a higher HbA1c (10.4 ± 3.0% vs. 9.8 ± 2.8%, p<0.001) and eGFR (123.1 ± 15.5ml/min/1.73m^2^ vs. 114.6 ± 19.3ml/min/1.73m^2^, p<0.001) at diagnosis (**Table 1**).

### Microvascular and macrovascular complications composite development following diagnosis

Of the 672 Ethiopian patients, 197 (29%) developed at least one microvascular complication compared with 3,937 (33%) of the non-Ethiopian patients.

Ethiopian patients who developed microvascular complications tended to be younger at diagnosis (37.5 ± 15.6 vs. 40.9 ± 17.4, p=0.001), had a significantly lower SES, and presented with higher HbA1c and eGFR levels at the index date. However, they had lower rates of hyperlipidemia, hypertension, and obesity (**Table 2**).

**Table 2:** Comparison of the non-Ethiopian and Ethiopian type 1 diabetes patients with microvascular complications.

Characteristics*	Non-Ethiopian (n=3,937)	Ethiopian (n=197)	P value**
Age at diagnosis T1D, years	40.9 ± 17.4	37.5 ± 15.6	0.001^a^
Males	2,193 (56)	105 (53)	0.508^b^
BMI, kg/m^2^	26.8 ± 5.4	23.7 ± 3.7	<0.001^a^
Missing	362 (9)	27 (14)	
Systolic BP, mmHg	123.0 ± 16.2	118.3 ± 18.0	0.002 ^a^
Missing	1,322 (34)	48 (24)	
Diastolic BP, mmHg	74.8 ± 9.9	73.2 ± 9.2	0.009 ^a^
Socioeconomic status:			**<0.001** ^b^
Low	2,036 (52)	151 (77)	
Medium	496 (13)	3 (2)	
High	1,034 (26)	36 (18)	
Missing	371 (9)	7 (4)	
Smoking status			**<0.001** ^b^
Never	1,931 (49)	123 (62)	
Current	817 (21)	28 (14)	
Former	670 (17)	14 (7)	
Missing	519 (13)	32 (16)	
Alcohol use	87 (2)	19 (10)	**<0.001** ^b^
Chronic diseases before, at, or after the index date			
Obesity	1,791 (46)	43 (22)	**<0.001** ^b^
Hypertension	2,052 (52)	88 (45)	**0.041** ^b^
Hyperlipidemia	1,492 (38)	51 (26)	**<0.001** ^b^
Laboratory results at the index date			
HbA1c, %	9.7 ± 2.7	10.3 ± 2.8	0.004^a^
Missing	541 (14)	13 (7)	
Total Cholesterol, mg/dL	209.5 ± 56.2	203.4 ± 52.5	0.043^a^
Missing	238 (6)	8 (4)	
LDL-Cholesterol, mg/dl	124.6 ± 40.7	122.6 ± 42.0	0.148^a^
Missing	711 (18)	22 (11)	
Triglycerides, mg/dL	224.3 ± 265.1	199.0 ± 166.4	0.273^a^
Missing	264 (7)	13 (7)	
Creatinine, mg/dL	0.9 ± 0.8	0.8 ± 0.3	<0.001^a^
Missing	180 (5)	5 (3)	
GFR, ml/min/1.73m^2^	109.3 ± 20.9	121.6 ± 17.0	<0.001^a^
Missing	1,508 (38)	45 (23)	
Albumin/creatinine ratio, mg/g	56.4 ± 202.7	63.9 ± 236.8	0.407^a^
Missing	2,016 (51)	92 (47)	

T1D, Type 1 diabetes; BMI, Body mass index; BP, blood pressure; HbA1c, Hemoglobin A1c; eGFR, Estimated glomerular filtration rate; LDL, Low-density lipoprotein; SD, Standard deviation.

*Data presents mean ± SD or n (%). **Patients non-Ethiopian vs Ethiopian; P-value is significant at <0.05.

a Two-sample t test.

b Chi-Square Test (χ²). Bold means the value has a statistical significance.

Additionally, Ethiopian patients with T1D were found to have a significantly lower incidence of macrovascular complications (7% vs. 16%, p<0.001). Overall, baseline characteristics were similar between the two groups, and differences followed the same pattern as described for the entire cohort. The mean age for macrovascular complications was higher than for microvascular complications in both groups (**Table 3**).

**Table 3:** Comparison of the non-Ethiopian and Ethiopian type 1 diabetes patients with macrovascular complications.

Characteristics*	Non-Ethiopian (n=1,947)	Ethiopian (n=50)	P value**
Age at diagnosis T1D, years	50.2 ± 14.4	48.2 ± 16.4	0.091^a^
Males	1,242 (64)	35 (70)	0.367^b^
BMI, kg/m^2^	27.3 ± 5.1	24.0 ± 3.5	**<0.001**
Missing	136 (7)	5 (10)	
Systolic BP, mmHg	127.8 ± 17.1	124.0 ± 14.8	0.141 ^a^
Missing	633 (33)	10 (20)	
Diastolic BP, mmHg	76.3 ± 10.4	74.3 ± 8.1	0.074 ^a^
Socioeconomic status:			
Low	1,072 (55)	35 (70)	**0.006** ^b^
Medium	307 (16)	0 (0)	
High	385 (20)	13 (26)	
Missing	183 (9)	2 (4)	
Smoking status			0.117 ^b^
Never	813 (42)	25 (50)	
Current	414 (21)	6 (12`)	
Former	445 (23)	8 (16)	
Missing	275 (14)	11 (22)	
Alcohol use	58 (3)	8 (16)	**<0.001** ^b^
Chronic diseases before, at, or after the index date			
Obesity	903 (46)	7 (14)	**0.001** ^b^
Hypertension	1,458 (75)	36 (72)	0.643 ^b^
Hyperlipidemia	1,271 (65)	26 (52)	0.052 ^b^
Laboratory results at the index date			
HbA1c, %	9.4 ± 2.6	10.2 ± 2.9	0.039^a^
Missing	272 (14)	5 (10)	
Total Cholesterol, mg/dL	215.5 ± 54.3	209.1 ± 43.5	0.401 ^a^
Missing	90 (5)	2 (4)	
LDL-Cholesterol, mg/dl	128.3 ± 36.7	122.7 ± 37.9	0.166 ^a^
Missing	90 (5)	3 (6)	
Triglycerides, mg/dL	243.1 ± 270.0	213.9 ± 201.3	0.127 ^a^
Missing	94 (5)	2 (4)	
Creatinine, mg/dL	1.0 ± 0.8	0.8 ± 0.3	0.005 ^a^
Missing	60 (3)	0 (0)	
GFR, ml/min/1.73m^2^	103.6 ± 21.1	115.1 ± 20.8	0.001 ^a^
Missing	668 (34)	9 (18)	
Albumin/creatinine ratio, mg/g	54.6 ± 170.4	37.1 ± 68.7	0.533 ^a^
Missing	1,005 (52)	22 (44)	

T1D, Type 1 diabetes; BMI, Body mass index; BP, blood pressure; HbA1c, Hemoglobin A1c; eGFR, Estimated glomerular filtration rate; LDL, Low-density lipoprotein; SD, Standard deviation.

*Data presents mean ± SD or n (%). **Patients non-Ethiopian vs Ethiopian; P-value is significant at <0.05.

a Two-sample t test.

b Chi-Square Test (χ²). Bold means the value has a statistical significance.

**Figure 2:**
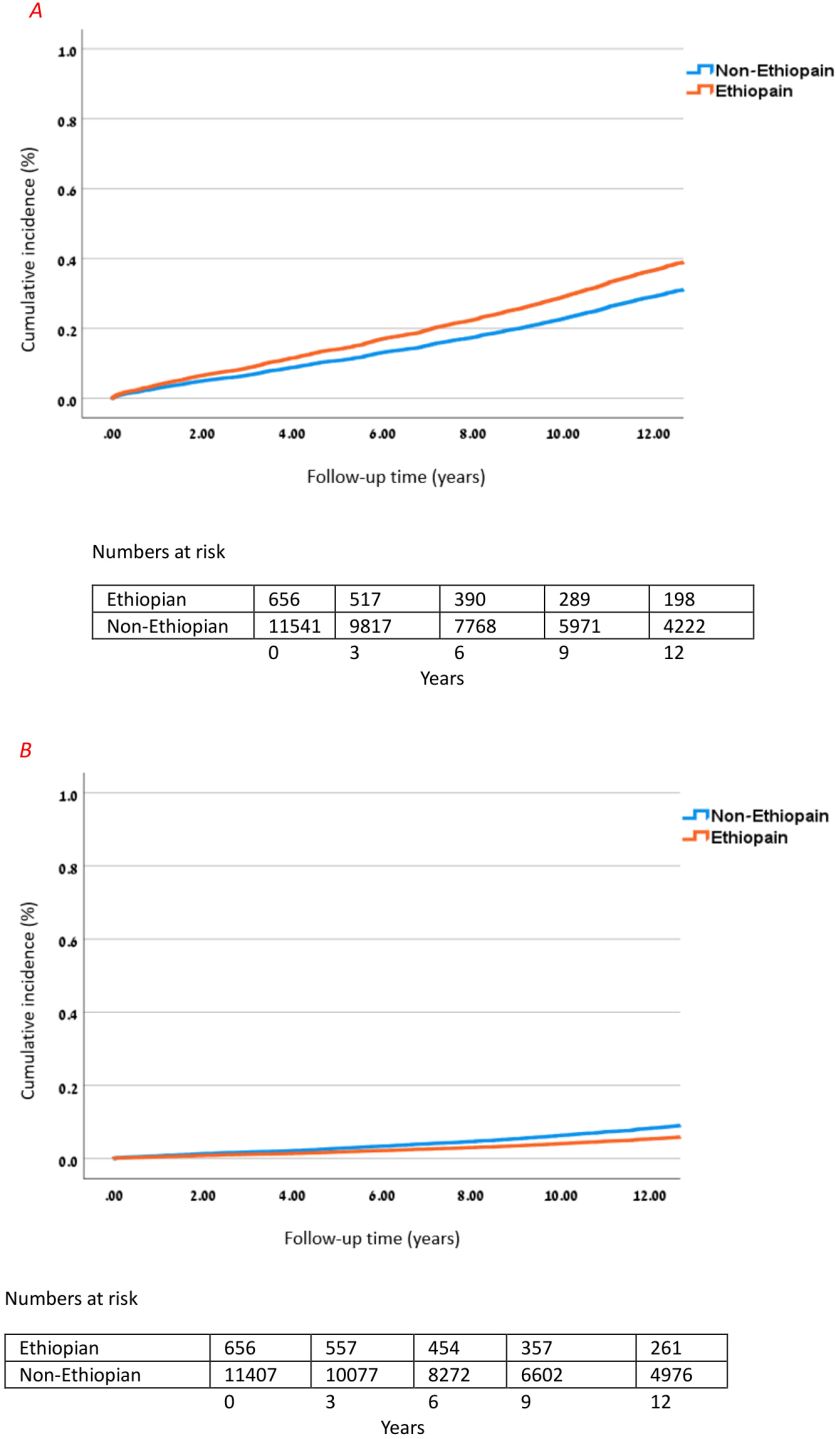
Cumulative incidence of microvascular and macrovascular complications in Ethiopian. **(A)** Cumulative incidence of microvascular complications in Ethiopian vs. non-Ethiopian T1D patients over 12 years. Cox model adjusted for age, sex, BMI, and HbA1c; HR for Ethiopian ethnicity vs. non-Ethiopian: 1.325 (95% CI 1.124–1.563), p = 0.001. **(B)** Cumulative incidence of macrovascular complications in Ethiopian vs. non-Ethiopian T1D patients over 12 years. Cox model adjusted for age, sex, BMI, and HbA1c; HR for Ethiopian ethnicity vs. non-Ethiopian: 0.606 (95% CI 0.425–0.863), p < 0.001.

In the multivariable Cox regression model for microvascular complications and macrovascular complications, Ethiopian ethnicity was independently associated with higher risk of developing the composite endpoint of any microvascular complication (adjusted HR 1.325; 95% CI 1.124–1.563; p = 0.001) and had a significantly lower risk of developing the composite of macrovascular complications (adjusted HR 0.606; 95% CI 0.425–0.863; p < 0.001) as compared to people of non-Ethiopian ethnicities. Other independent risk factors in the adjusted model included age at T1D diagnosis (HR 1.017 per year; 95% CI 1.014–1.019; p < 0.001) and (HR 1.045 per year; 95% CI 1.041–1.049; p < 0.001); male gender (HR 1.113; 95% CI 1.035–1.198; p = 0.004) and (HR 1.753; 95% CI 1.560–1.969; p < 0.001); BMI (HR 1.030 per kg/m2; 95% CI 1.023–1.037; p < 0.001) and (HR 1.037 per kg/m2; 95% CI 1.026–1.048; p < 0.001); and elevated HbA1c (HR 1.086 per %; 95% CI 1.072–1.101; p < 0.001) and (HR 1.062 per %; 95% CI 1.038–1.085; p < 0.001) for microvascular and macrovascular complications, respectively (**Table 4** and **Figure 2**).

**Table 4:** Adjusted hazard ratios from multivariable Cox regression analyses of variables associated with microvascular and macrovascular complications in non-Ethiopian and Ethiopian type 1 diabetes patients.

Characteristics	Microvascular complications			Macrovascular complications		
	HR	95% CI	P-value	HR	95% CI	P-value
Ethiopian ethnicity	1.325	1.124-1.563	**0.001**	0.606	0.425-0.863	**<0.001**
Age at diagnosis of T1D	1.017	1.014-1.019	**<0.001**	1.045	1.041-1.049	**<0.001**
Males	1.113	1.035-1.198	**0.004**	1.753	1.560-1.969	**<0.001**
BMI	1.030	1.023-1.037	**<0.001**	1.037	1.026-1.048	**<0.001**
HbA1c	1.086	1.072-1.101	**<0.001**	1.062	1.038-1.085	**<0.001**

HR, Hazard ratio; CI, Confidence Interval; BMI, Body mass index; T1D, Type 1 diabetes; HbA1c, Hemoglobin A1c. Bold means the value has a statistical significance.

### Sensitivity analyses

When using attained age as the time scale, Ethiopian ethnicity remained associated with a lower hazard of incident macrovascular complications (HR 0.57; 95% CI 0.40–0.81; p = 0.002) after adjustment for sex, BMI, and HbA1c. In a sensitivity model further adjusting for smoking, obesity, and SES, the estimate was directionally consistent and of similar magnitude but borderline for statistical significance (HR 0.68; 95% CI 0.46–1.01; p = 0.057), likely reflecting reduced power due to missingness in the additional covariates. HbA1c, male sex, smoking, and obesity were each independently associated with higher macrovascular risk, whereas SES was not.

Using attained age as the time scale, the association between Ethiopian ethnicity and lower macrovascular hazard was consistent in direction across age: 18–39 years (HR 0.43; 95% CI 0.20–0.92; p = 0.029), 40–59 years (HR 0.74; 95% CI 0.46–1.18; p = 0.203), and ≥60 years (HR 0.51; 95% CI 0.24–1.08; p = 0.079). With extended adjustment (adding smoking, obesity, SES), estimates were similar in magnitude but less precise (e.g., 40–59 years (HR 0.61; 95% CI 0.36–1.03; p = 0.063).

The protective association persisted across risk strata and was significant among participants with ≥2 risk factors in the primary model: 0 risk factors (HR 0.43; 95% CI 0.14–1.36; p = 0.149), 1 risk factor (HR 0.53; 95% CI 0.27–1.04; p = 0.066), ≥2 risk factors (HR 0.59; 95% CI 0.38–0.93; p = 0.022). In extended models (adding smoking and SES), the estimates remained protective but were not statistically significant, consistent with reduced power and potential over−adjustment for covariates on the causal pathway.

Findings were similar when restricting to age at entry ≥40: primary model (HR 0.61; 95% CI 0.41–0.92; p = 0.017), extended model (HR 0.71; 95% CI 0.45–1.13; p = 0.149).

Collectively, these analyses suggest that the lower macrovascular hazard observed in Ethiopian patients is not fully attributable to age distribution or a lower baseline burden of classical cardiovascular risk factors and remains directionally robust across clinically relevant strata (**Supplementary Table 2**).

## Discussion

In this study, we examined the incidence of composite microvascular and macrovascular complications among individuals with T1D of Ethiopian and non-Ethiopian ethnicities. The findings reveal striking and divergent risk patterns. In Cox proportional hazards models, individuals of Ethiopian descent were 30% more likely to develop microvascular complications, highlighting a concerning vulnerability, yet were 40% less likely to experience macrovascular complications, underscoring a markedly different risk profile compared to their non-Ethiopian counterparts.

We also demonstrate differences in some baseline characteristics between the two populations. While age at diagnosis was similar between groups, people of Ethiopian ethnicity were more likely to be of male sex, with a lower BMI and a higher HbA1c level at diagnosis, and a lower incidence of hypertension and hyperlipidemia. These differences may account for the differences in complication patterns.

To this end, clinical guidelines do not provide different recommendations for follow-up or treatment of microvascular and macrovascular complications in T1D patients among different ethnicities. Guidelines from the American Diabetes Association (ADA)/European Association for the Study of Diabetes (EASD) recommend that management and surveillance strategies should be based on individual risk factors, glycemic control, and comorbidities; however, they do not stratify risk based on ethnicity (13, 14). Importantly, guidelines acknowledge the disparities in complication rates and outcomes among ethnic groups, but state that current evidence is insufficient to support ethnicity-specific clinical recommendations, and that observed differences may be confounded by SES, genetics, and other risk factors rather than requiring different clinical approaches (15).

Recent studies, such as those from the UK Biobank and National Health and Nutrition Examination Surveys (NHANES), have highlighted ethnic disparities in T1D outcomes, including differences in prevalence, genetic risk factors, and glycemic control. Integrating these global perspectives enhances the understanding of ethnic variations in T1D (16, 17).

Other observational studies confirmed that certain ethnic groups may carry higher or lower risks for specific diabetes-related complications (18, 19). Similar to our findings, Mangelis et al. demonstrate in a UK-based study that Afro-Caribbean ethnicity was a risk factor for kidney disease in T1D patients, showing faster kidney function deterioration and more than double the incidence of advanced kidney disease compared to other ethnic groups (6). Conversely, in a systematic review evaluating ethnic differences in T1D outcomes, South Asian patients demonstrated higher cardiovascular mortality and a less favorable metabolic profile compared to White Europeans, while rates of microvascular complications were largely comparable. These findings suggest that the well-documented ethnic disparities in macrovascular outcomes seen in type 2 diabetes (T2D) may also be present among individuals with T1D (20).

Several studies conducted in Ethiopia point to different pathogeneses that may explain the differences. Studies on T1D conducted in different regions in Ethiopia found distinct patterns of disease behavior and reported that more than 70% of children and adolescents with T1D had poor glycemic control and showed high rates of microvascular complications in patients with relatively short duration of disease and at a young age (21–23).

Furthermore, there is increasing evidence that the clinical presentation of T1D in Ethiopia differs from the “classical” presentation in affluent countries, concerning age of onset, altered clinical phenotype, and different autoantibody profiles (24–26). A recent epidemiological study on 1682 new onset T1D cases who presented to the diabetes clinics of the Gondar area in Ethiopia over 20 years (1996–2016) with a rapid onset of classical diabetes symptoms and treated with insulin from the first clinic visit, suggest that the common disease pattern differs from that of western countries with a later age of onset and a very low incidence of T1D in childhood (3.3% diagnosed under 15 years) followed by a rapid increase in onset among the 25-29 years old group, a male predominance at presentation and a lower prevalence of indicators of islet-cell autoimmunity (26).

The same group of researchers investigated 236 cases presenting clinically with T1D at the Gondar regional clinics. They demonstrated that most of the Ethiopians had a single autoantibody (usually anti-GAD) compared to multiple autoantibodies in the European group. Additionally, autoantibody positivity in Ethiopians decreased more rapidly with increasing age of onset compared to Europeans; importantly, both groups were tested shortly after clinical diagnosis (22).

Relatively little is known about the clinical outcomes of T1D in Ethiopia, and most studies conducted had small sample sizes. In the study by Eshetu et al., 9.7% of patients aged under 18 were found to have mild non-proliferative retinopathy, while 11.2% had diabetic kidney disease (23). The median time for microvascular complications was seven years post-diagnosis, and an increased risk of microvascular complications was noted among male patients (23). According to Hintsa et al., the prevalence of kidney disease in adult patients rose by 43% at 10 years after diagnosis (27). Limited studies from Ethiopia demonstrated a wide range of complications, with the prevalence of retinopathy ranging from 5% to 38.5%, nephropathy from 15.7% to 29.5%, and peripheral neuropathy from 16.4% to 82.7% (23, 27–30).

In our study, we similarly observed an increased incidence of microvascular complications among Ethiopian patients, further supporting concerns regarding the burden of microvascular disease in this population. Ethiopian patients who developed microvascular complications had significantly lower SES and presented with higher HbA1c and elevated eGFR levels, suggestive of early hyperfiltration at the index date compared to the rest of the cohort. All of these factors are well-established risk factors for the subsequent development of microvascular complications (27–30).

Additionally, the differences in the phenotypic characteristics and manifestation of T1D in non-Ethiopian and Ethiopian populations may be due to the complex interplay of environmental factors, including lifestyle, dietary habits, education, SES, and limited access to healthcare. Less frequent medical visits among the Ethiopian population may negatively impact glycemic control and, as a result, contribute to the progression of diabetic complications.

A single study from Ethiopia investigated the rate of macrovascular complications in T1D. During a median follow-up of 5.9 years, 11 (3.9%) of 118 patients with a mean age of 43.9 ± 15.8 years developed cardiovascular disease (CVD). Three risk factors for CVD were identified: kidney disease, increased systolic BP, and increased triglycerides (31).

Ethiopian males had a higher incidence of T1D than females, exhibited significantly lower BMI, and a reduced prevalence of abnormal triglyceride levels, total cholesterol, and systolic BP. These metabolic and cardiovascular parameters are among the strongest predictors of CVD risk, which may partly explain the lower incidence of diabetic macrovascular complications in Ethiopian patients compared to their non-Ethiopian counterparts.

The major strengths of this study are the availability of longitudinal data and the large sample size. Additionally, this is the first study to undertake phenotypic characterization of T1D in adult Ethiopian Israeli patients to investigate if ethnicity-related differences exist in the incidence of diabetes-related complications. Data was recorded from the largest health maintenance organization in Israel, and we used rigorous criteria to capture T1D patients. To our knowledge, this is the first study to thoroughly investigate the incidence of diabetes-related complications in an adult T1D Ethiopian population and to make comparisons with a non-Ethiopian Israeli population.

Our study has several limitations. The presented data is based on retrospective EMR data that has biases and missing information regarding the type of diabetes. This may have led to under-capture of late-onset autoimmune T1D and over-inclusion of insulin-treated T2D in young adults. To minimize the impact of this limitation on the population, we have restricted the study sample by strict criteria, eliminating the majority of members with T2D from our sample.

Our study did not include data on genetic markers or autoantibody profiles, and this lack of immunogenetic characterization limits the ability to provide a mechanistic interpretation of the observed differences between Ethiopian and non-Ethiopian patients with T1D. Another limitation is that the data does not contain information regarding environmental factors, including lifestyle and dietary habits, previously shown to be associated with diabetes-related complication development. As we relied mostly on ICD-10 codes for diagnosis, we cannot rule out the possibility of underreporting in the Ethiopian population with lower SES and fewer clinic visits. The possibility that we didn’t capture all events cannot be ruled out. Additionally, follow-up duration was insufficient to capture differences among the Ethiopian population already born in Israel with a shorter duration of diabetes, compared to the previous generation of immigrants. Complete-case analysis may have reduced statistical power and introduced selection bias if missingness was related to outcomes or unmeasured confounders, as suggested by the attenuation of significance in extended models. Finally, we used maximum laboratory values within the baseline window to capture peak metabolic exposure, which has prognostic significance for diabetes complications. While this conservative approach prevents underestimation from early treatment effects or regression to the mean, it may introduce detection bias if testing frequency differs between Ethiopian and non-Ethiopian populations. Future studies using alternative definitions (closest value, mean) could validate our findings.

In conclusion, despite significant advancements in T1D management over the past decades, diabetic complications remain a major concern. Our findings emphasize the multifactorial nature of these complications and the need for targeted interventions in high-risk populations. Additionally, they highlight the role of ethnicity in diabetes-related outcomes, suggesting that Ethiopian ethnicity may be associated with a distinct complication pattern and a need for closer surveillance concerning microvascular complications. Further data is needed to establish whether there is a need for ethnic- and population-specific therapeutic and management approaches for T1D. Understanding these differences is crucial for optimizing care and improving long-term outcomes. Further research is essential to uncover the underlying mechanisms driving these disparities and to develop tailored strategies for diabetes management across diverse populations.

## Data Availability

The raw data supporting the conclusions of this article will be made available by the authors, without undue reservation.
