# Supplementary material for: Distinct complication profiles: a comparative study of Ethiopian and non-Ethiopian adults with type 1 diabetes
Source: Front Endocrinol (Lausanne). 2025 Oct 17;16:1664230. doi: 10.3389/fendo.2025.1664230 (PMC12575150; doi:10.3389/fendo.2025.1664230)
Supplement: Supplementary file 1 [file Table1.docx]

*Supplemental Table 1.* *Codes used for defining diagnosis and outcomes according to ICD-10 codes*

| **Category** | **Diagnosis** | **Code** |
| --- | --- | --- |
| **Outcome** |  |  |
| Microvascular complications | Retinopathy | E10.3, E10.6, E10.8, E11.3, E11.6, E11.8, H36.0 |
|  | Nephropathy | E10.2, E10.6, E10.8, E11.2, N18.1, N18.2, N18.3, N18.6, N18.9, N19, R80.9, Z94 |
|  | Neuropathy | E10.4, G63.2 |
| Macrovascular complications | Ischemic heart disease | I24.9, I25.1, I25.3 I25.5, I25.6, I25.7, I25.8, I25.9 |
|  | Myocardial infarction | I21, I22, I24, I25.2 |
|  | Chronic heart failure | I50, I50.1, I50.2, I50.3, I50.4, I50.8, I50.9, I11.0 |
|  | Peripheral vascular disease | E10.5, I70.2, I73, I73.8, I73.9, I79.2 |
|  | Cerebrovascular accident | I63, I63.9, I64, I67.2-4, I67.8-9, I69, G46, Z86.73 |
|  | Transient ischemic attack | G45, Z86.73 |
|  | Carotid artery disease | I70.8 |
| **Chronic disease** |  |  |
|  | Alcohol | F10 |
|  | Smoking | F17, F17.20, F17.210, Z72.0, Z87.891 |
|  | Hypertension | I10 |
|  | Hyperlipidemia | E78.0-5 |
|  | Obesity | E66.0, E66.9, Z68.3 |
|  | Metabolic associated liver disease | K75.81 |
| **Exclusions diagnosis** |  |  |
|  | Gestational diabetes mellitus | 024.4, 024.8, 024.9 |
|  | Neonatal diabetes mellitus | P70.2 |
|  | Post pancreatectomy diabetes mellitus | E13 |
|  | Pancreatitis, postprocedural diabetes mellitus and other diseases of the pancreas | K85-86 |
