# Supplementary material for: Distinct complication profiles: a comparative study of Ethiopian and non-Ethiopian adults with type 1 diabetes
Source: Front Endocrinol (Lausanne). 2025 Oct 17;16:1664230. doi: 10.3389/fendo.2025.1664230 (PMC12575150; doi:10.3389/fendo.2025.1664230)
Supplement: Supplementary file 2 [file Table2.docx]

*Supplementary Table 2. Association between Ethiopian ethnicity and macrovascular composite outcomes using attained-age Cox*

| ***Model specification*** | ***N*** | ***Events*** | ***HR (95% CI)*** | ***P value*** |
| --- | --- | --- | --- | --- |
| ***Attained-age models*** | | | | |
| *Primary attained-age model (adjusted for sex, BMI, HbA1c)* | *8,363* | *1,280* | *0.568 (0.399–0.809)* | ***0.002*** |
| *Extended attained-age model (additionally adjusted for smoking, obesity, SES)* | *6,635* | *992* | *0.679 (0.456–1.012)* | *0.057* |
| ***Age-stratified models*** | | | | |
| *18–39 years (primary)* | *2,997* | *241* | *0.43 (0.20–0.92)* | ***0.029*** |
| *18–39 years (extended)* | *2,395* | *198* | *0.54 (0.24–1.24)* | *0.145* |
| *40–59 years (primary)* | *1,657* | *525* | *0.74 (0.46–1.18)* | *0.203* |
| *40–59 years (extended)* | *1,285* | *406* | *0.61 (0.36–1.03)* | *0.063* |
| *≥60 years (primary)* | *489* | *213* | *0.51 (0.24–1.08)* | *0.079* |
| *≥60 years (extended)* | *388* | *163* | *0.63 (0.26–1.57)* | *0.325* |
| *<18 years* | *1,491* | *13* | *–* | *–* |
| ***Risk-profile stratified models*** | | | | |
| *0 risk factors (primary)* | *3,564* | *104* | *0.43 (0.14–1.36)* | *0.149* |
| *0 risk factors (extended)* | *2,721* | *80* | *0.63 (0.20–2.04)* | *0.444* |
| *1 risk factor (primary)* | *2,798* | *288* | *0.53 (0.27–1.04)* | *0.066* |
| *1 risk factor (extended)* | *2,133* | *220* | *0.52 (0.23–1.19)* | *0.120* |
| *≥2 risk factors (primary)* | *1,959* | *888* | *0.59 (0.38–0.93)* | ***0.022*** |
| *≥2 risk factors (extended)* | *1,474* | *690* | *0.77 (0.47–1.27)* | *0.308* |
| ***Restriction analysis*** | | | | |
| *Age ≥40 years (primary)* | *4,145* | *1,090* | *0.61 (0.41–0.92)* | ***0.017*** |
| *Age ≥40 years (extended)* | *3,182* | *835* | *0.71 (0.45–1.13)* | *0.149* |

*HR: Hazard ratio; CI: Confidence Interval; BMI: Body mass index; T1D: HbA1c: Hemoglobin A1c; SES: Socioeconomic status.*

*Primary model adjusted for sex, BMI, and HbA1c. Extended model additionally adjusted for smoking, obesity, and SES. Risk-factor strata defined as count of hypertension, hyperlipidemia, and obesity at cohort entry.*
